# Cardiovascular magnetic resonance (CMR) imaging of the aorta in pregnancy: imaging and outcome

**DOI:** 10.1186/1532-429X-14-S1-T14

**Published:** 2012-02-01

**Authors:** Jane M Francis

**Affiliations:** 1 Oxford Centre for Clinical Magnetic Resonance Research, University of Oxford, Oxford, UK; 2 The John Radcliffe Hospital, Oxford, UK

## Summary

Women with genetic cardiovascular diseases,or repaired congenital defects require additional monitoring during pregancy and may need planned surgical delivery of the infant. CMR is a simple, accurate multi parametric imaging techniques which provides assessment of cardiac anatomy, function and flow in the pregnant patient.

## Background

A retrospective analysis of the cardiac and obstetric notes of 5 women was carried out to evaluate the use of CMR, the need for further imaging/intervention and outcome of the pregnancy.

## Methods

5 women aged 22-30 (mean 26.6 years) were referred for CMR during 1st (3) pregnancy and 2nd pregnancy(2). Patients were 16-28 weeks pregnant(mean 22 weeks) at the time of imaging Previous medical history and indications for CMR were: severe aortic stenosis and bicuspid valve with syncopal episodes (1), Marfans syndrome with chest pain, aortic dissection on trans oesophageal echo (1), aortic coarctation and increasing aortic root dimensions on trans thoracic echocardiography (1), aortic coarctation and bicuspid aortic valve, patent ductus arteriosus and ventricular septal defect presenting with chest pain (1) and investigation of a cardiac source of emboli causing ischaemic episodes in right leg (1).Imaging was carried out on a 1.5T Siemens Sonata or Avanto ( Ehrlagen, Germany) using a spine array posteriorly and 2 channel flex array placed over the anterior chest. Patients were placed supine then a wedge was placed under the right hip to minimise aorto-caval compression. All images were acquired using ECG gating and sequences limited to answer the clinical question. Acquisitions were kept at normal operating mode with an specific absorption rate (SAR) of 2w/kg. Imaging protocol comprised SSFP (steady state free precession) localisers in three orthogonal planes; anatomical assessment in axial, coronal and sagittal/sagittal planes using HASTE (Half -Fourier acquisition single shot turbo spin echo) long and short axis SSFP retrospectively gated breathold cines for the visual assessment of ventricular function. Short axis images were acquired for accurate analysis of ventricular function and mass . Phase shift velocity mapping was added for the evaluation of flow and peak velocity in patients with valvular disease or aortic coarction.

## Results

All patients tolerated CMR- average imaging time of 25mins. 4 patients delivered at 34-37 week (mean 35 weeks) 3 planned caesarean sections,1 spontaneous vaginal delivery.Birth weight 2370-3076 grams (mean 2589 grams).Balloon valvuloplasty was carried out within 1 week of CMR in the patient with severe aortic stenosis and surgical aortic valve replacement 3 months post delivery. No further CMR has been performed. No evidence of dissection found in patient with Marfans syndrome but aortic root was dilated(4.1 cm) which remained stable 2 months post delivery and 3 years later. No cardiac cause of emboli was found in the patient with right leg ischaemic episodes and no cause for chest pain or recurrence of re-coarctation seen in the remaining patients and no further CMR has been performed.

## Conclusions

CMR is a safe reliable imaging method for the diagnostic assessment of the aorta and cardiac function in pregnancy It can be used as a screening tool pre, during and post pregnancy and can guide intervention and aid planning delivery.

## Funding

Jane Francis is funded by OCMR- Oxford Centre for Clinical Magnetic Resonance Research, University of Oxford.

